# Efficient Sulfonation of Syndiotactic Polystyrene Membranes Assessed by Small‐Angle Neutron Scattering

**DOI:** 10.1002/cplu.202500735

**Published:** 2026-04-18

**Authors:** Aurel Radulescu, Hiroki Iwase, Shin‐ichi Takata

**Affiliations:** ^1^ Jülich Centre for Neutron Science at Heinz Maier‐Leibnitz Zentrum Forschungszentrum Jülich GmbH Garching Germany; ^2^ Comprehensive Research Organization for Science and Society CROSS Neutron Science and Technology Centre Tokai Japan; ^3^ Materials and Life Science Division Japan Proton Accelerator Complex J‐PARC Tokai Japan

**Keywords:** contrast variation, host–guest systems, hydrophilic membranes, neutron scattering, semicrystalline polymers

## Abstract

The selectivity of the δ‐form of syndiotactic polystyrene (sPS) toward small organic molecules enables homogeneous functionalization by sulfonation of sPS membranes via soaking in a chloroform solution of a bulky sulfonating agent such as lauroyl sulfate. Sulfonated syndiotactic polystyrene (s‐sPS) is hydrophilic and shows a high proton conductivity comparable to that of Nafion, which is the benchmark in proton‐exchange membrane fuel cell technology. Therefore, s‐sPS might represent a good candidate for the hydrocarbon alternative to the hazardous fluorinated compounds such as Nafion in various energy conversion applications. However, for optimal performance, functionalization should be efficient across the entire volume of the membrane. Here, we report on the evaluation of the efficiency of sulfonation of sPS membranes based on a semiquantitative analysis of data measured by small‐angle neutron scattering on dry and hydrated samples. By exploiting the different response of neutrons from the same compound with different degrees of deuteration, various structural details were highlighted following in‐beam hydration of functionalized membranes with H_2_O or D_2_O. Experimental analysis of the scattering data under different contrast conditions using the one‐dimensional correlation function Γ_1_(*x*) provided evidence of homogeneous sulfonation of only the amorphous phase of the membranes, while the crystalline regions remained unaffected.

## Graphical abstract

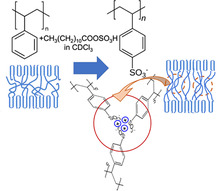

## 1 Introduction

Syndiotactic polystyrene (sPS) is a relatively new material [1] that exhibits some very interesting properties such as (i) a complex polymorphic behavior, including five different crystalline forms in which the polymer chains either adopt a planar zigzag (α‐ and β‐forms) or a TTGG helix (γ‐, δ‐, and ε‐forms) conformation [2, 3, 4], and (ii) the ability to form various types of cocrystalline (clathrate) phases with a wide range of small organic molecules that can be incorporated as guests into the cage‐ or channel‐like cavities between the polymer helices of the δ‐ or ε‐forms [5, 6, 7]. Depending on the solvent used, helical cocrystalline δ‐ and ε‐forms can be produced either by solvent‐induced crystallization in cast films or by exposing amorphous films to solvents in vapor or liquid state. Moreover, the initial guest molecules in sPS cocrystals can be smoothly replaced by other molecules through exposure to vapors or liquids of new solvents [8, 9]. The clathrate forms are particularly attractive for applications where active guests can be incorporated into the sPS films, enabling them advanced materials for optical and magnetic applications [10, 11]. Furthermore, the emptied clathrates, obtained through appropriate guest extraction methods [12], can be used as molecular sieves [13] for water purification of chlorinated hydrocarbons.

The modification of sPS by attaching sulfonate groups along the polymer backbone with the aim of making the material hydrophilic and thus ion‐conductive upon hydration was investigated in some early studies, which were motivated by the good chemical resistance and mechanical stability of the material [14, 15]. Several sulfonation procedures of sPS from solution have been proposed, using either acetyl sulfate or hexanoyl sulfate as sulfonating agents and chloroform, 1,2,4‐trichlorobenzene (TCB) or 1,1,2‐trichloroethane (TCE) as solvents. Sulfonation was reported to significantly change the crystallization properties of the polymer. As the degree of sulfonation increased, the melting point, degree, and rate of crystallization were lowered due to the introduction of sulfonic acid groups. More recently, a solid‐state sulfonation method for semicrystalline sPS films has been proposed that takes advantage of the selectivity of the sPS δ‐form for hydrocarbon solvents and yields homogeneous functionalization of sPS films up to 200 µm in thickness without a deterioration of the crystalline phase [16]. The idea is to use a bulky sulfonating agent that has a molecular volume much larger than the maximum volume of 250 Å^3^ of possible guest molecules that can be incorporated into the sPS clathrate phase [17]. By immersing the sPS films in a chloroform solution of lauroyl sulfate, (i) the film samples are sulfonated quickly and evenly across the entire film thickness, since chloroform can easily penetrate the crystalline phase, and (ii) while the large molecule of the sulfonating agent cannot penetrate the clathrate δ‐form, sulfonation only takes place in the amorphous regions of the film, thereby preserving the crystallinity of the sample. It has been observed that procedures involving sulfonating agents with smaller molecules, which can also penetrate the crystalline δ‐form, cause deterioration of the crystalline phase [18]. The degree of sulfonation (DS), expressed as S atoms/styrene units x 100 mol%, can be controlled by varying the concentration of the reagent in chloroform solution or the time the polymer films are exposed to the reagent. Sulfonated syndiotactic polystyrene (s‐sPS) is hydrophilic and at high DS shows a high proton conductivity comparable to that of Nafion [19, 20, 21], which is the benchmark in proton‐exchange membrane (PEM) fuel cell technology [22]. Moreover, hydration–drying cycles were not having a disruptive effect on the crystalline phase, as observed by small‐angle neutron scattering (SANS) [23]. Therefore, s‐sPS might represent a good candidate for the hydrocarbon alternative to the hazardous fluorinated compounds such as Nafion in the proton‐exchange membrane applications. However, the effective application of s‐sPS type membranes in the energy field is not yet matured and further studies on improving their chemical stability and conductive properties under specific temperature (T) and relative humidity (RH) conditions are still required.

Another aspect that requires special attention is the question of whether sulfonation actually occurs across the entire thickness of the membrane or whether there is a highly sulfonated surface and nonsulfonated areas. Depth profile analysis using SEM–EDS reported a high and nearly constant degree of sulfonation across the entire thickness of sPS films with a crystalline phase characterized by the δ‐form [17]. However, SEM–EDS can only provide very local information and no global information over the entire length of the film. To verify the global results of the sulfonation process, an analysis of the sample volume is required. Thermogravimetric analysis (TGA) [24, 25] and neutron prompt gamma activation analysis (PGAA) [23, 26] provide information that is averaged over the sample volume. TGA is a destructive method that provides information about the thermal stability and composition of the sample by analyzing the multistep decomposition profile at increasing temperatures. However, due to the superposition of signals from different decomposition processes, the interpretation of the results can be difficult in some cases for functionalized polymers [27]. Furthermore, TGA cannot provide information about the distribution of functional groups and must often be supplemented by X‐ray diffraction (XRD) and differential scanning calorimetry analyses for a more precise characterization of the sample. PGAA is a nondestructive method that can also provide information about the composition averaged over the sample volume [28], but does not provide results about the distribution of the various components in the sample. Thus, none of these methods can evaluate the efficiency of sulfonation throughout the membrane both qualitatively (distribution) and quantitatively (amount) and mainly provide global quantitative information averaged over the entire sample volume.

SANS is a powerful technique for investigating structures and morphologies in a broad length scale from 10 to 1000 Å. As neutral particles, neutrons have a large penetration depth. Furthermore, at energies corresponding to the wavelengths typically used in SANS experiments (*λ* in the range 3–7 Å), neutrons are not producing sample beam damage. As a consequence, SANS can provide averaged structural information about bulk samples with a thickness typically up to a few millimeters. Moreover, samples can be studied under variable environmental conditions such as humidity and temperature using complex ancillary equipment. However, the unique advantage of neutron scattering methods stems from the ability of neutrons to see light atoms in the presence of heavier ones and to distinguish between the isotopes of the same element. These properties enable the use of isotope substitution methods to highlight structural and dynamic details in a complex sample. Particularly, the large difference in cross section between hydrogen (H) and deuterium (D) enables the contrast variation (CV) methods to be used in the investigation of synthetic macromolecules and biomaterials [29]. Generally, in a neutron scattering experiment the visibility of a scattering object embedded in a medium depends on the so‐called contrast factor that is the difference of the object/medium scattering length densities. The ability to vary the scattering contrasts between different constituents of a multicomponent hydrocarbon sample over a broad range by selective H–D substitution allows the neutron scattering of the sample to be separated into that from components [30]. CV is particularly strong for resolving the structure and morphology of complexes in solution, when the D labeling can be done either within the complex (internal CV [31]) or in solvent (external or solvent CV [32]). However, the same can be applied in the characterization of partially hydrophilic hydrocarbon membranes: the contrast between the hydrophilized domains and the rest of the polymer membrane can be varied over a broad range by using H_2_O/D_2_O at various ratios for hydrating the membrane, to highlight or mask different structural features. A typical example is that of the Nafion membranes, which were thoroughly characterized by CV–SANS [33]. Furthermore, geological materials can as well be structurally characterized in details using CV–SANS with penetrating fluids, which allows measurement of the size range of accessible versus inaccessible pores [34].

SANS and neutron diffraction have been used to study gelation of sPS in various solvents: D labeling of different components helped understanding the sPS chain conformation in solution and gel state [35, 36]. Furthermore, SANS with D labeling was used in experiments on solvent‐induced crystallization and guest exchange in sPS [9]. Also, we have recently demonstrated how using selective deuteration in SANS and wide‐angle neutron scattering (WANS) experiments at the same beamline can be used for a successful and thorough structural and morphological characterization of PEMs such as those made from s‐sPS [26].

Here, SANS is proposed as a nondestructive method for more accurately evaluating the efficiency of sulfonation of sPS membranes. The contrast between the crystalline lamellae and the functionalized (hydrophilic) amorphous layers was varied by using either H_2_O or D_2_O to hydrophilize the functionalized interlamellar layers. This allowed scattering details to be masked or highlighted depending on the degree of deuteration of the samples, the degree of sulfonation, and the hydration of the membrane. A detailed structural characterization was performed by interpreting the scattering data using the one‐dimensional correlation function, while the efficiency of membrane sulfonation was evaluated by semiquantitative interpretation of the scattering intensity as a function of the contrast between different components of the membrane.

## 2 Results and Discussion

Scattering techniques explore matter in reciprocal space and the information about the structure and morphology of the investigated sample is contained in the scattering intensity *I*(*Q*) measured as a function of the momentum transfer *Q*, with *Q* = (4*π*/*λ*) sin*θ*, where *λ* is the neutron wavelength and 2*θ* is the scattering angle [29]. *Q* acts as a kind of inverse yardstick: large *Q* values relate to short distances, while a small *Q* relates to large size objects. Thus, crystalline arrangement of atoms and molecules, which are typically characterized by sizes and correlation lengths of a few Å, are investigated from the analysis of scattering at high *Q*, via wide‐angle X‐ray scattering (WAXS) and wide‐angle neutron scattering (WANS). On the other hand, association of molecules yielding large‐scale objects such as macromolecules, micelles, lamellae, vesicles or, generally, a phase separation in the sample, are characterized following the observation of scattering at small angles (small‐angle X‐ray scattering (SAXS) and SANS). Typically, the scattering of semicrystalline polymers, which consist of a mixture of amorphous and crystalline phases, yields distinct features as follows. (i) In the *Q* range covered by SAXS and SANS experiments (typically from 0.001 to 0.1 Å^–1^), the signals originate from the crystalline lamellae, the interlamellar correlation (“long spacing”) due to the difference in scattering length density (SLD) between the stacked crystalline lamellae and the amorphous interlamellar layers, and the inhomogeneities on a scale of several to hundreds of nanometers in the amorphous phase; (ii) in the high *Q* range covered by the WAXS and WANS data (≈1 Å^–1^), the crystalline reflections corresponding to the specific lattice of the crystalline phase of the polymer occur. These scattering signals are averaged over all possible orientations of the constituent morphologies in a cast film, so that a superposition of different scattering signals is measured in a specific *Q* range. In order to separate the signals of different morphologies from each other, especially in the small‐angle scattering range, uniaxially deformed films should be used for structural analysis.

**FIGURE 1:**
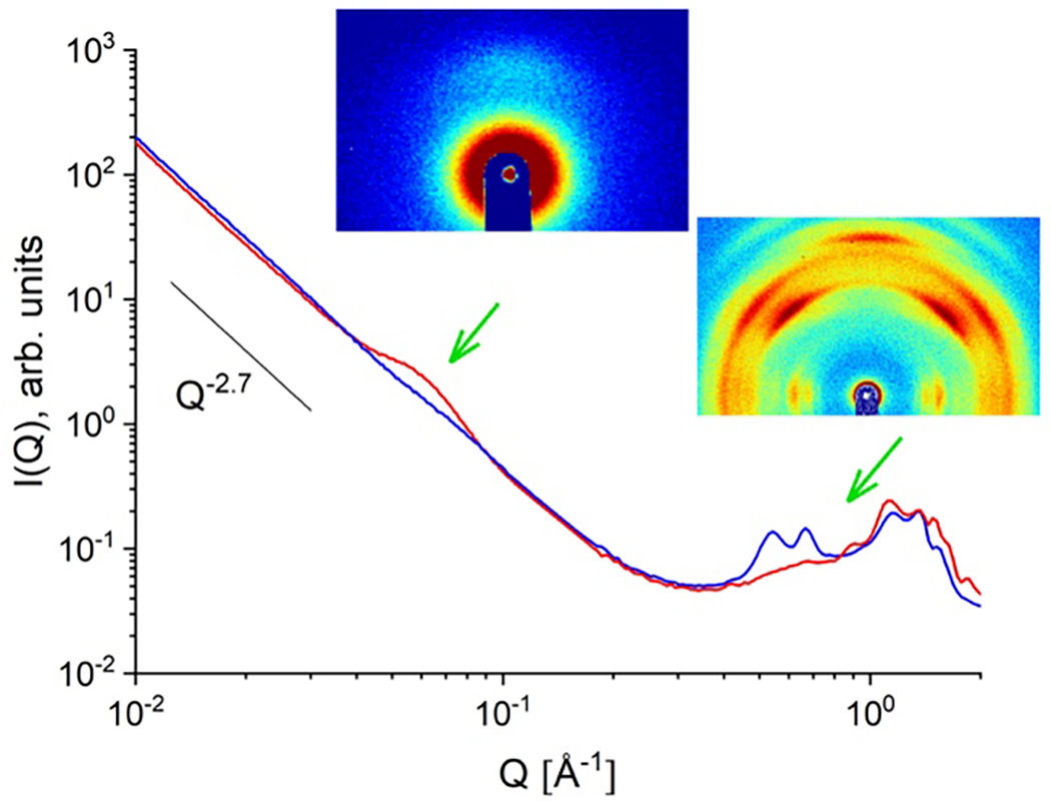
Typical SAXS and WAXS scattering patterns of a uniaxially deformed sPS film, both as two‐dimensional data and averaged over narrow detection sectors (±10°) along (meridian, red curve) and perpendicular (equator, blue curve) to the deformation axis of the film. The film was measured in an experimental geometry with a vertically positioned deformation axis in beam. SAXS = Small‐angle X‐ray scattering; WAXS = wide‐angle X‐ray scattering.

Figure 1 shows typical scattering patterns obtained by SAXS and WAXS from a uniaxially deformed sPS film (deformation factor 2.5) containing a cocrystalline phase (clathrate) with toluene molecules as guests in the cavities between the sPS helices in the crystalline lamellae. The interlamellar correlation peak appears in the meridian sectors when the film is positioned with the deformation axis vertical in the beam. This is due to the alignment of the crystalline lamella stacks along the deformation direction. The peak position *Q*_lam_ ≈ 0.07 Å^–1^ results in a long spacing *L*_lam_ ≈ 90 Å, while the broad character of the peak indicates a relatively broad distribution of *L*_lam_. A monotonic decrease in scattering intensity with increasing *Q* is observed over the entire SAXS range in the equatorial sectors. Such a feature is observed for many amorphous polymers, for example, atactic polystyrene (aPS) [37]. The monotonic power law behavior of the scattering intensity shown in Figure 1, which behaves like *I*(*Q*) ~ *Q* ^−*p*^ at low *Q*, is due to scattering at structural heterogeneities that occur in the amorphous phase of membranes at different length scales. The exponent *p* ≈ 2.7 is very close to that observed in the case of aPS [37]. An attempt has been made to interpret such power‐law behavior of the scattering intensity in terms of the mass‐fractal character of the structural heterogeneities, although its nature is still unclear [37].

The arcs observed with WAXS are due to the orientation of different crystal planes in the crystal lamellae that are aligned across the direction of deformation. The WAXS pattern at high *Q* is used to identify the crystal forms of sPS and determine the crystal structure. The two sharp reflections observed in the equatorial sectors between *Q* = 0.5 and 0.7 Å^–1^ indicate a δ‐form with helical sPS chains and a high amount of guest molecules stored in the cavities between the sPS helices [38].

Unfortunately, X‐ray scattering cannot provide precise information about the position and distribution of different types of molecules in a complex sample. This is particularly disadvantageous in the case of time‐resolved structural/morphological changes due to the in‐beam application of external fields or composition changes [39], or in the case of the functionalized amorphous phase of sPS films by sulfonation and subsequent exposure of the film sample to variable relative humidity and temperature. In such cases, information about the density distribution of the molecules during guest exchange and the structure of the functionalized domains in the sample can be obtained by neutron scattering. Neutron scattering is characterized by coherent and incoherent scattering contributions, which depend on the scattering properties of the individual nuclei [40]. Coherent scattering is characterized by the coherent scattering length *b*_coh_ and leads to interference between neutrons scattered by different nuclei. This provides information about the structure of the scattering system, which is contained in the *Q*‐dependent scattering features that observed in the scattered intensity *I*(*Q*). Incoherent scattering, characterized by the incoherent scattering length *b*_incoh_, contains no structural information, but only information about the scattering material density, and appears in the SANS/WANS pattern as a *Q*‐independent constant background. To describe the scattering of atomic groups (nuclei) such as macromolecules or molecular assemblies, where the relevant length scales are much larger than the atomic dimensions, the scattering length density distribution SLD ρ(r) is used. This corresponds to the total scattering length of the atoms per unit volume. For a given compound (molecule), the SLD can be expressed as ρ = $\sum_{i}^{n}b_{i}/V_{m}$, where *b*_i_ is the (coherent) scattering length of the atom (nucleus) “i” in the compound and *V*_m_ is the molecular volume of the compound. Small‐angle scattering arises as a result of inhomogeneities in the SLD. For simple systems, such as water domains formed in a hydrophilic polymer matrix, the difference in SLD or the contrast Δρ between the matrix and the domains controls the magnitude of the measured scattering intensity *I*(*Q*)

(1) $$I\left(Q\right)=\text{φ Δ}{\text{ρ}}^{2}\;V\;P(Q)\;S\left(Q\right)+\text{Bckgd}$$

where φ is the volume fraction of hydrated domains in the sample, *V* is the volume of the hydrated domains, *P*(*Q*) is the form factor that depends on the size and shape of the hydrated domains, and *S*(*Q*) is the structure factor, which describes the correlation effects between the domains. At a low volume fraction when hydrated domains are well separated from each other, thus *S*(*Q*) = 1. The second term in Equation (1) represents the background, which is mainly caused by various incoherent scattering signals from the sample. A prerequisite for a successful SANS/WANS neutron scattering experiment is therefore the maximization of the signal‐to‐background ratio during the experimental structural analysis. This can be achieved by manipulating the composition of a sample through H or D labeling of different components, exploiting the large difference between *b*_coh_ of H (–0.3741 × 10^–12^ cm) and D (0.6671 × 10^–12^ cm). Since the incoherent scattering length of H (*b*_incoh_ = 2.5274 × 10^–12^ cm) is much larger than that of D (*b*_incoh_ = 0.404 × 10^–12^ cm) [40], the D label not only allows for a variation in neutron contrast, but also minimizes the incoherent background. Since the molecules affected by the H/D exchange are chemically identical, the physical chemistry of the samples is altered only slightly, if at all.

**FIGURE 2:**
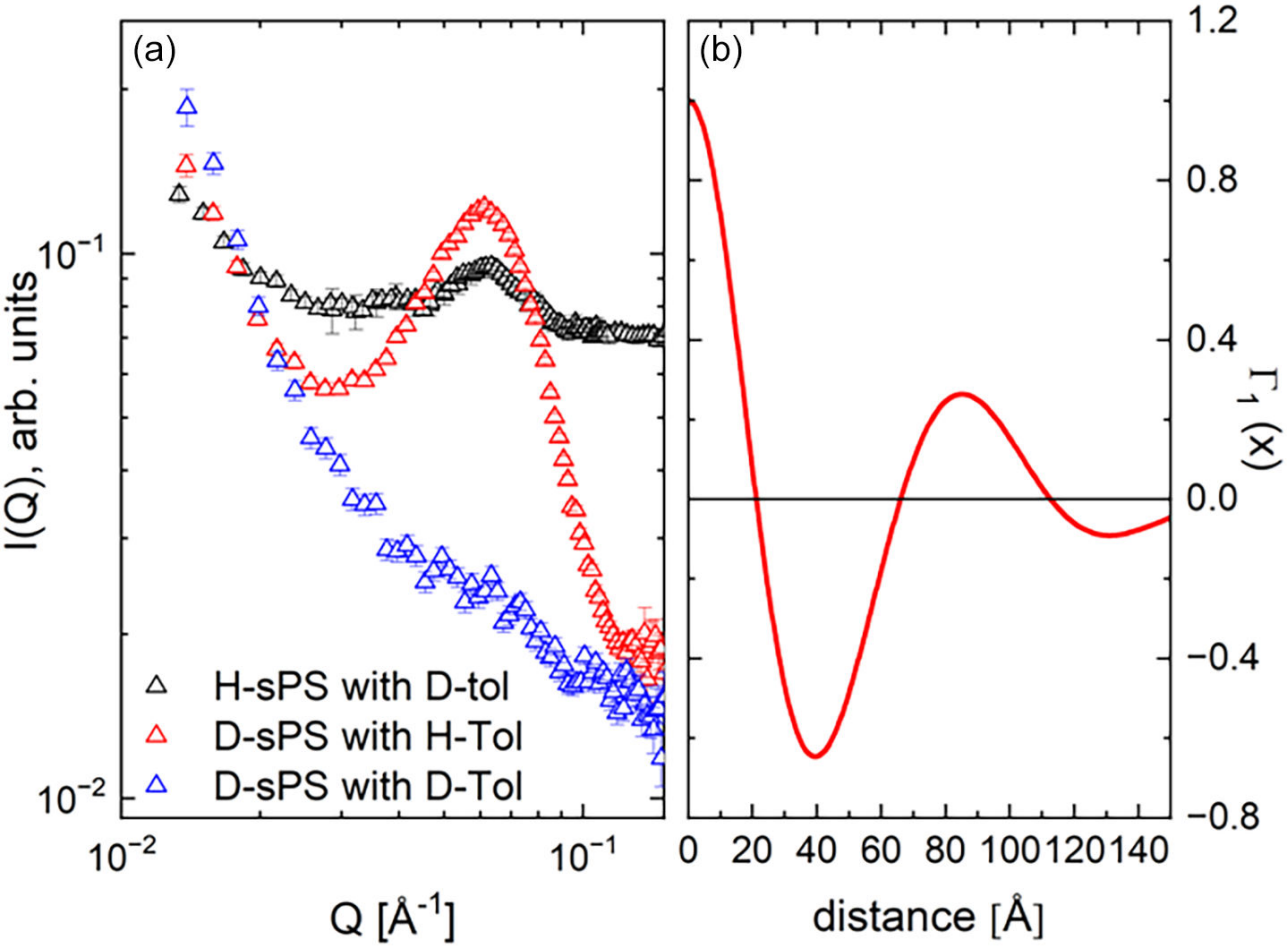
Typical SANS patterns (a) of a uniaxially deformed sPS film under different contrast conditions, after averaging over narrow detection sectors (±10°) along the deformation axis of the film, and the one‐dimensional correlation function Γ_1_(*x*) (b), calculated from the experimental data under the most favorable contrast conditions (red symbols in field a). SANS = Small‐angle neutron scattering.

Figure 2a shows the SANS results of uniaxially deformed sPS films loaded with toluene molecules as guests in the cocrystalline δ‐form. The H/D degree of the sample was varied in different ways. Considering an sPS film in the hydrogenated state (H‐sPS) loaded with deuterated toluene molecules (D‐Tol) as guests in the cavities between the sPS helices (black symbols in Figure 2a), the high incoherent background level from the hydrogenated sPS film results in a weak signal‐to‐background ratio for the interlamellar correlation peak. A much lower background is obtained when working with D‐sPS films. This enables greater sensitivity in enhancing or suppressing the interlamellar correlation peak by changing the contrast between the crystalline lamellae loaded with toluene guest molecules and the interlamellar amorphous layers. This can be achieved by varying the state of the guest molecule in H or D form. When using H‐Tol, a higher contrast between the crystalline lamellae and the amorphous D‐sPS interlamellar layers enhances the interlamellar correlation effect (red symbols in Figure 2a). When D‐Tol is used, the contrast between the crystalline lamellae and the amorphous interlamellar layers, both of which are deuterated, is very low, causing the interlamellar correlation peak to disappear (blue symbols in Figure 2a). The one‐dimensional correlation function Γ_1_(*x*), which was derived by Fourier transformation of the scattering intensity under the maximum contrast condition in Figure 2a, is shown in Figure 2b. When interpreting Γ_1_(*x*) according to the approach proposed by Strobl, Schneider, and Voigt‐Martin [41], a lamella thickness *d*_lam_ = 32 Å and a long spacing *L*_lam_ = 83 Å are obtained for the crystalline phase of the sPS semicrystalline film sample, while a film crystallinity of 36% was derived, which was similar to that obtained from powder XRD.

**FIGURE 3:**
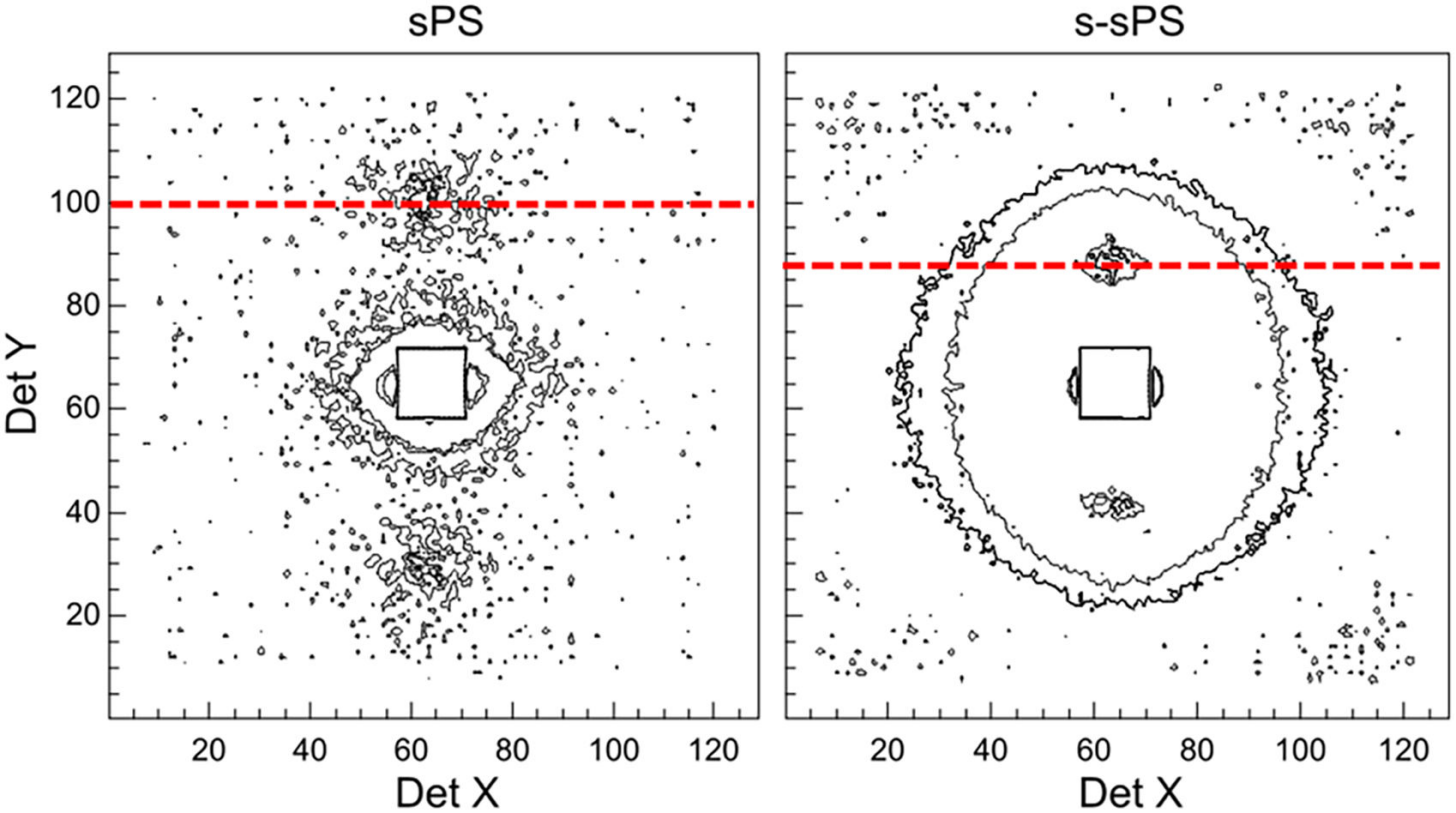
Two‐dimensional scattering patterns from the uniaxially deformed sPS films containing the δ crystalline form before (left) and after sulfonation (right). The red dotted lines indicate the position of the interlamellar peak that appears at smaller scattering angles after the film sulfonation.

Sulfonation of the sPS film induces changes in the film structure. These changes can be better observed by comparing the two‐dimensional scattering patterns of the unsulfonated and sulfonated, uniaxially deformed films (Figure 3). In both cases, the interlamellar correlation peak is observed in the meridian sectors, although in the case of the sulfonated film it occurs at smaller scattering angles compared to the nonsulfonated film. This indicates that the sulfonated films are characterized by a larger long spacing in the crystalline phase. In addition, a new morphological level appears in the sulfonated film, leading to isotropic scattering, which can be observed around the beam stop at scattering angles greater than the position of the interlamellar peak (Figure 3, right). It is known that sulfonated head groups of the polymer side chains undergo cluster‐like aggregation, an effect that has been well studied in the case of Nafion [42, 43] and sulfonated PEEK [44]. The ion clusters have an inverted micelle structure and sizes of several tens of Å, depending on the polymer type. As previously reported [23, 26], the additional isotropic scattering observed in the scattering patterns of s‐sPS films is due to the presence of ionic aggregates (clusters). These clusters grow in size and connect with each other during membrane hydration and are responsible for the ionic conductivity in such membranes. For the purposes of the present study, however, only the meridian sectors of the scattering patterns, as shown in Figure 3, will be further analyzed and discussed.

**FIGURE 4:**
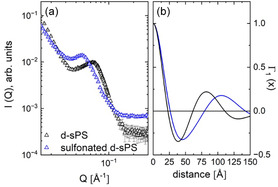
SANS patterns (a) of a uniaxially deformed sPS film before (black symbols) and after (blue symbols) sulfonation, obtained by averaging over narrow detection sectors (±10°) along the deformation axis of the film. The panel (b) shows the one‐dimensional correlation function Γ_1_(*x*) derived from the experimental data in panel (a).

Figure 4a shows the SANS patterns recorded for uniaxially deformed sPS and sulfonated sPS films (s‐sPS) and averaged over the sectors along the deformation axis. The aggregation of sulfonated groups in the interlamellar layers leads to an increase in the long spacing in the functionalized membrane to *L*_lam_ = 106 Å, while the lamellar thickness remained constant as in the nonfunctionalized membrane, *d*_lam_ = 32 Å, as can be seen from the interpretation of the Γ_1_(*x*) function derived from the experimental data along the deformation axis (Figure 4b). This indicates that sulfonation had no effect on the crystalline phase, while the width of the interlamellar layers increased from ≈51 to 74 Å due to the formation of sulfonated clusters after functionalization of the sample.

**FIGURE 5:**
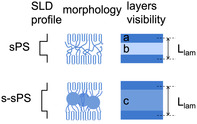
Schematic presentation of the morphologies derived from the analysis of the data in Figure 4, including the SLD profile across the lamellar stack and the “visibility” of different layers in the lamellar stack. Layer a is the crystalline lamella, layer b is the amorphous interlamellar layer, and layer c is the sulfonated amorphous layer, with the ionic aggregation clusters indicated in the morphology. The visibility scheme resulted from the consideration of a homogeneous SLD across each layer.

A schematic representation of the morphologies discussed based on the data shown in Figure 4 is presented in Figure 5. Due to the difference in SLD between the crystalline lamellae (layer a) and the amorphous interlamellar layers (layers b), as shown by the SLD profile shown in Figure 5, the interlamellar correlation effect was visible in the scattering data, and *L*_lam_ was extracted from the analysis of the Γ_1_(*x*) function.

The sulfonation of the membrane and the formation of ion clusters led to swelling of the amorphous layer (layer c). The difference in SLD between the crystalline and sulfonated layers allowed the observation of a larger *L*_lam_ compared to the nonsulfonated case. The effect of sulfonation was quantitatively verified by subjecting the sulfonated membrane to in situ hydration during the SANS experiment. A relative humidity of RH = 80% was achieved using either H_2_O or D_2_O vapors to vary the SLD of the functionalized interlamellar layer in the beam.

**FIGURE 6:**
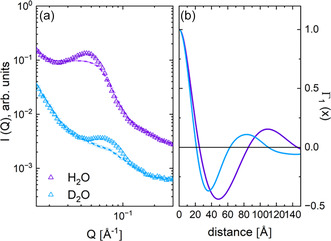
SANS patterns (a) from the uniaxially deformed sPS film sulfonated at a low DS and in situ hydrated at RH = 80% (30°C) with either H_2_O or D_2_O vapors, after averaging the data over narrow detection sectors (±10°) along (symbols) and perpendicular (dotted) the film deformation axis, and the one‐dimensional correlation function Γ_1_(*x*) derived from the experimental data (b). SANS = Small‐angle neutron scattering.

The scattering patterns along the deformation axis of the hydrated membrane with a low DS = 13.5% are shown in Figure 6a. Hydration of interlamellar layers with H_2_O yields a much higher scattering intensity than that of the dry membrane (Figure 4a), which indicates a larger contrast between the hydrated interlamellar layers and the crystalline lamellae. The hydration of sample with D_2_O results in a lower scattering intensity than that from the dry membrane, which means a lower contrast between the hydrated interlamellar layers and the crystalline lamellae compared to the dry membrane. It appears that the interlamellar correlation peak is also affected by the variation in contrast, as its position occurs at a higher *Q* value under the hydration condition with D_2_O than when using H_2_O. From the analysis of the function Γ_1_(*x*) (Figure 6b) for the hydrated cases, we can conclude that *d*_lam_ remains rather constant (33 and 30 Å for the samples hydrated with H_2_O and D_2_O, respectively), while *L*_lam_ is 110 and 82 Å in the two cases, respectively. The *L*_lam_ of the membrane hydrated with H_2_O is slightly larger than that of the dry sulfonated membrane, which is due to slight swelling of the interlamellar layers upon water absorption. Under similar hydration conditions, i.e., at constant relative humidity, and if hydration occurs homogeneously, the width of the interlamellar layer should remain the same regardless of whether H_2_O or D_2_O is used. However, a much smaller *L*_lam_ value was obtained for the membrane hydrated with D_2_O. To understand this observation, the scattering intensities between the peaks detected under different hydration conditions can be interpreted in terms of the contrast between different layers, as described in the Supporting Information (SI). The rationalization regarding the effects of the contrast factor on the observed scattering intensities applies well when comparing the dry film and the film hydrated with H_2_O. Hydration of the sulfonated interlamellar layer with D_2_O causes its SLD to approach that of the crystalline lamellae, so that the interlamellar correlation peak should be barely visible. Under these contrast condition, however, a weaker but distinct correlation peak is observed at higher *Q* values. The peak position corresponds fairly well with that of the interlamellar peak observed for the nonsulfonated membrane (Figure 4a). Two conclusions can be drawn from this observation. First, the hydration of the sulfonated interlamellar layers with D_2_O closely matched out the correlation effect between the lamellae aligned along the deformation axis in the functionalized lamellar stacks. Second, at this low DS of membrane, there still appears to be a fraction of the interlamellar layers that were not functionalized during the sulfonation process. The nonfunctionalized interlamellar layers remain hydrophobic and cause the weak scattering peak shown in Figure 6a for the D_2_O contrast condition (light blue symbols). When the same membrane is hydrated with H_2_O, this peak, which occurs due to interlamellar correlations in the nonfunctionalized lamellar stacks, is much weaker and is therefore masked by the strong peak due to the correlation effect between the layers in the hydrated regions of the membrane.

**FIGURE 7:**
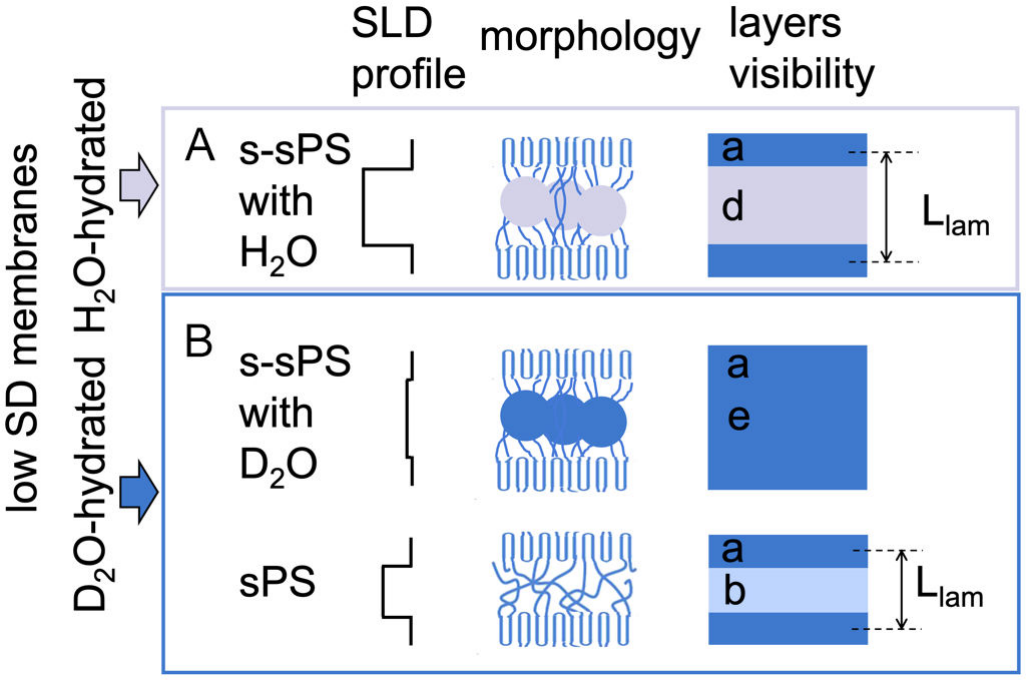
Schematic presentation of the morphologies derived from the analysis of the data in Figure 6, including the SLD profile across the lamellar stack and the “visibility” of different layers in the lamellar stack. Layer a is the crystalline lamella, layer b is the amorphous interlamellar layer (nonsulfonated layer), layer d is amorphous interlamellar layer containing ionic clusters that were hydrated with H_2_O, and layer e is amorphous interlamellar layer containing ionic clusters that were hydrated with D_2_O. The visibility scheme resulted from the consideration of a homogeneous SLD across each layer. The hydration with D_2_O led to matching out the interlamellar correlation effect, rendering the effect “invisible.”

Membranes with low DS values therefore contain sulfonated and nonsulfonated interlamellar layers. The different nature of the peaks observed for the hydration conditions H_2_O and D_2_O suggests that the scattering pattern shown in Figure 6a results from the superposition of signals from hydrated and nonsulfonated morphologies. An interpretation of the scattering intensities as a function of the contrast between different layers under the hydration conditions with H_2_O and D_2_O is again presented in detail in the SI. According to these results for the membrane with a low DS value, 57.6% of the interlamellar layers are sulfonated and hydrated, while the rest are unsulfonated. Again, Figure 7 presents a schematic representation of the discussed morphologies based on the data shown in Figure 6. Due to the large difference in SLD between the crystalline lamellae (layer a) and the sulfonated interlamellar layers hydrated with H_2_O (layer d), the interlamellar correlation peak was clearly visible in the scattering data. On the other hand, the SLD between the crystalline lamellae and the sulfonated interlamellar layers hydrated with D_2_O (layer e) is almost identical. As a result, the interlamellar correlation effect is not visible for the D_2_O‐hydrated portions of the membrane. However, the nonsulfonated areas of the membrane still exhibit an SLD difference between the crystalline and amorphous layers, making the correlation effect visible in the nonfunctionalized areas of the membrane.

**FIGURE 8:**
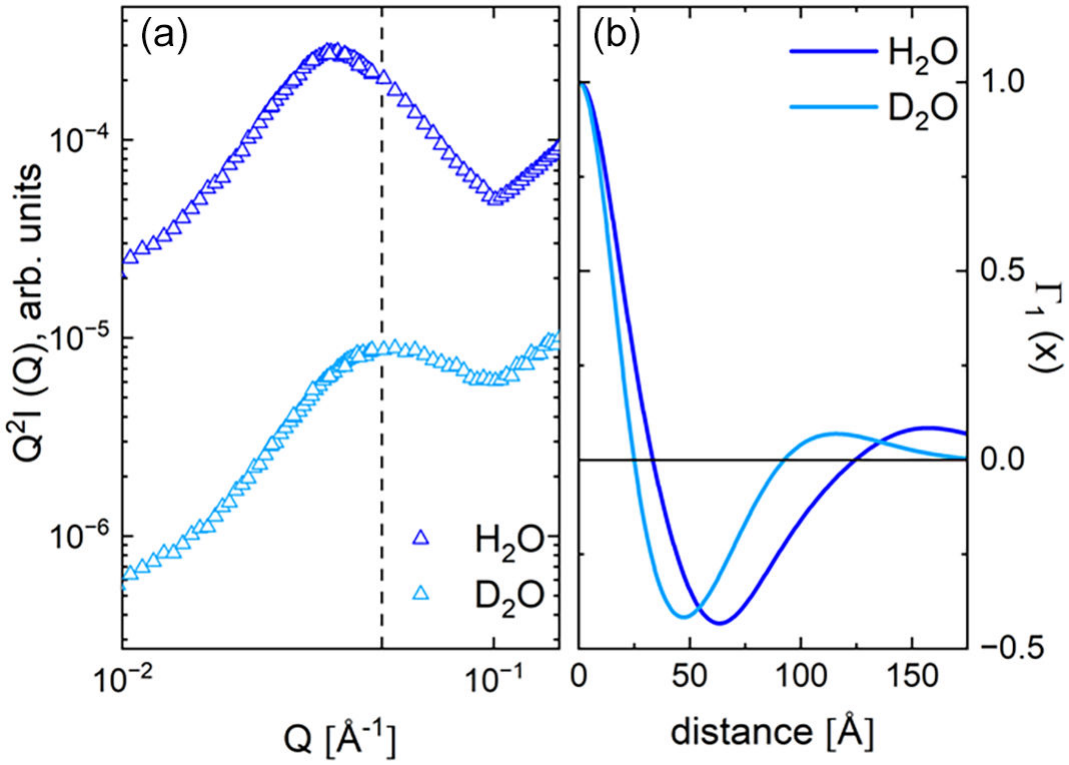
SANS patterns in a Kratky‐type plot (a) from the uniaxially deformed sPS film sulfonated at a high DS and in situ hydrated at RH = 80% (30°C) with either H_2_O or D_2_O vapors, after averaging the data over narrow detection sectors (±10°) along the film deformation axis, and the one‐dimensional correlation function Γ_1_(*x*) derived from the experimental data (b). SANS = Small‐angle neutron scattering.

**FIGURE 9:**
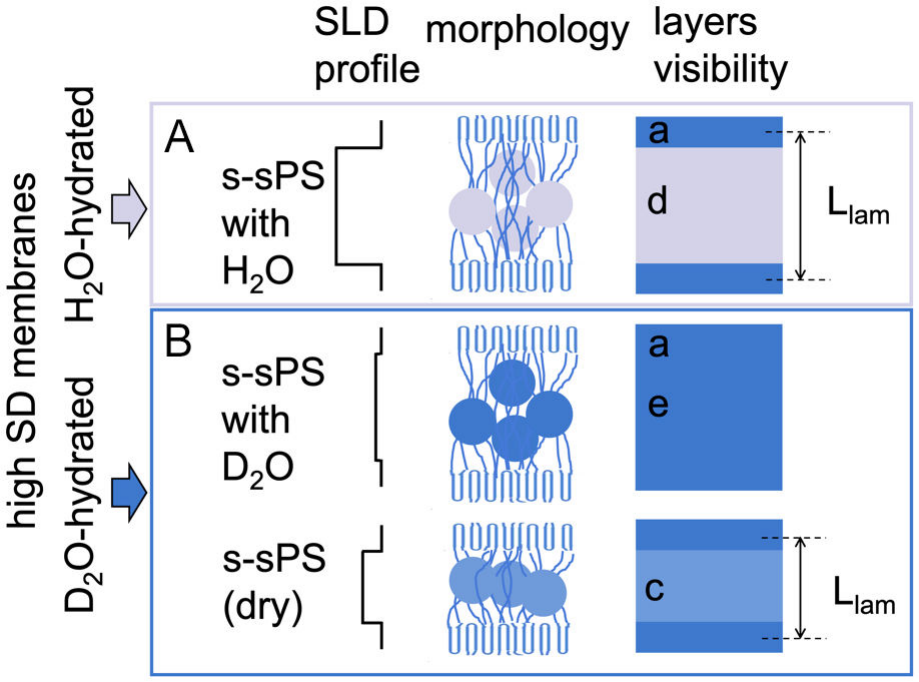
Schematic presentation of the morphologies derived from the analysis of the data in Figure 8, including the SLD profile across the lamellar stack and the “visibility” of different layers in the lamellar stack. Layer a is the crystalline lamella, layer c is the sulfonated amorphous interlamellar layer containing ionic clusters (dry layer), layer d is amorphous interlamellar layer containing ionic clusters that were hydrated with H_2_O, and layer e is amorphous interlamellar layer containing ionic clusters that were hydrated with D_2_O. The visibility scheme resulted from the consideration of a homogeneous SLD across each layer. The hydration with D_2_O led to matching out the interlamellar correlation effect, rendering the effect “invisible.”

The case of a film with a high sulfonation degree DS = 36%, which was hydrated with either H_2_O or D_2_O at a relative humidity of 80%, is shown in Figure 8. The SANS data averaged over the meridian sectors are shown in a Kratky plot to highlight the interlamellar correlation peak (Figure 8a). The data clearly show that the maximum of the interlamellar correlation peak occurs at a lower Q when the membrane is hydrated with H_2_O compared to the case where D_2_O was used. The Γ_1_(*x*) function (Figure 8b) shows that when the film was hydrated with D_2_O vapors, a shorter long spacing *L*_lam_ = 115 Å was observed than under the H_2_O contrast conditions, where the long spacing *L*_lam_ = 152 Å. For such highly sulfonated membranes, water uptake is ≈50%. This is defined as *W*_up−take_ = [(*W*_wet_ − *W*_dry_)/*W*_dry_], where *W*_wet_ and *W*_dry_ are the wet and dry weights of the membrane, respectively. Due to the high DS value and high water uptake, there is significant swelling of the interlamellar layer. For this membrane, a larger *L*_lam_ value is measured under H_2_O contrast conditions than for the membrane with a lower DS value under the same contrast conditions (Figure 6a). Interpretation of the Γ_1_(*x*) function for the H_2_O contrast condition in Figure 8b yields a value of *d*_lam_ = 41 Å, which is greater than the typical value of *d*_lam_ = 33 Å observed in the samples discussed previously. We can only speculate that this is an overestimation due to the interpretation of the scattering pattern resulting from uneven water distribution in the interlamellar layer. This would cause the thickness of the nonhydrated layers to appear greater than the effective thickness of the crystalline layer. As explained above, the D_2_O contrast condition is unfavorable for highlighting the interlamellar correlation effect. The Γ_1_(*x*) features for the D_2_O contrast condition are similar to those of the functionalized dry membrane (blue symbols and line in Figure 4). Therefore, the data interpretation indicates that in the D_2_O contrast condition there is a partition of the interlamellar layers into hydrated (matched out) and sulfonated but not hydrated (scattering‐producing) layers. As previously reported [45], not all ion clusters formed by aggregation of sulfonated groups appear hydrated in similar membranes examined at RH = 80%. A schematic representation of the morphologies discussed based on the data shown in Figure 8 is presented in Figure 9, together with the SLD between the layers in different hydration conditions and the layers “visibility” in different contrast conditions.

SANS provided averaged information over the entire sample volume, and the hydrated domains were either visible (under H_2_O contrast conditions) or matched out (under D_2_O contrast conditions). In the latter case, due to the limited hydration conditions (RH = 80%), there remained functionalized interlamellar layers that were still dry and caused the observed scattering. From the data interpretation, we therefore conclude that the sulfonation of the film is evenly distributed throughout the entire sample volume.

## 3 Experimental Section

Hydrogenated syndiotactic polystyrene was purchased from Idemitsu. The deuterated syndiotactic polystyrene was synthesized using d8‐styrene monomers (purchased from Sigma‐Aldrich, isotopic purity 98 at.% D), and a homogeneous catalytic system composed of pentamethylcyclopentadienyltitanium trichloride (purchased from Strem Chemicals) and methylalumoxane (purchased from Chemtura) in toluene (purchased from Sigma‐Aldrich). Amorphous films were prepared by melt pressing at 280°C followed by quenching at 0°C in an ice/water mixture. Oriented sPS deuterated and hydrogenated films were obtained by stretching the amorphous films with an INSTRON 4301 dynamometer at 110°C at a speed of 10 mm/min up to a draw ratio of about 2.5. The films were subsequently exposed to toluene vapors to obtain δ‐form clathrate samples. The state of the guest molecules incorporated in the cavities between the helical chains was changed between H and D forms by involving the guest exchange mechanism [9]. A part of the films containing the crystalline δ‐form was sulfonated via the solid‐state sulfonation procedure involving the lauroyl sulfate solution in CDCl_3_. The films characterization before and after sulfonation by powder XRD and single‐crystal XRD for the crystallinity and orientation of crystalline phase, by FTIR and TGA for the composition, and by PGAA for the sulfonation degree DS was described elsewhere [26].

Structural investigation of the H‐sPS and D‐sPS films containing cocrystalline δ‐form with various toluene guest molecules was carried out at the KWS‐2 pinhole SANS diffractometer [46] of Jülich Centre for Neutron Science, JCNS, in Garching, Germany, with a wavelength *λ* = 5 Å, while films in the clathrate, sulfonated, and hydrated states were studied at the time‐of‐flight SANS diffractometer TAIKAN (BL‐15) [47] at Japan Proton Accelerator Complex, J‐PARC, Japan, using a broad wavelength band *λ* from 0.8 to 7.8 Å. In situ film hydration and in‐beam water contrast variation between H_2_O and D_2_O vapors were achieved using a neutron scattering dedicated dew‐point generator and humidity chamber [48]. Details about the experiments and data reduction procedure are reported elsewhere [26]. In the current structural analysis, data measured over the wave‐vector transfer range *Q* between 0.01 and 0.3 Å^–1^ were used. Additional scattering features appear at much higher *Q* values due to sulfonation and hydration effects and are characterized by a much lower scattering intensity as the currently investigated scattering details. Therefore, in the current analysis a constant background was considered at high *Q* without affecting the quality of the obtained results. Preliminary SAXS–WAXS measurement on the uniaxially deformed sPS film containing δ cocrystalline form with toluene molecules was conducted at the Anton Paar SAXS point 5.0 diffractometer of J‐PARC, Tokai, Japan, using a Mo‐source and small‐ and wide‐angle detectors. The Γ_1_(*x*) function was obtained and interpreted using SasView5 package [49].

## 4 Conclusion

The functionalization of syndiotactic polystyrene membranes by sulfonation makes the material hydrophilic. The selectivity of sPS toward small organic molecules allows homogeneous functionalization of the membranes by soaking them in a chloroform solution of a bulky sulfonating agent such as lauroyl sulfate. For optimal performance, functionalization should be carried out efficiently over the entire volume of the membrane. Here, we report a semiquantitative analysis method for assessing the sulfonation efficiency on different sPS membranes using SANS in different contrast conditions. Membranes with varying degrees of sulfonation were analyzed under dry and hydrated conditions using in situ humidification and exchange of H_2_O or D_2_O vapors to create optimal conditions for the variation of neutron scattering length density between different regions of the membrane.

**FIGURE 10:**
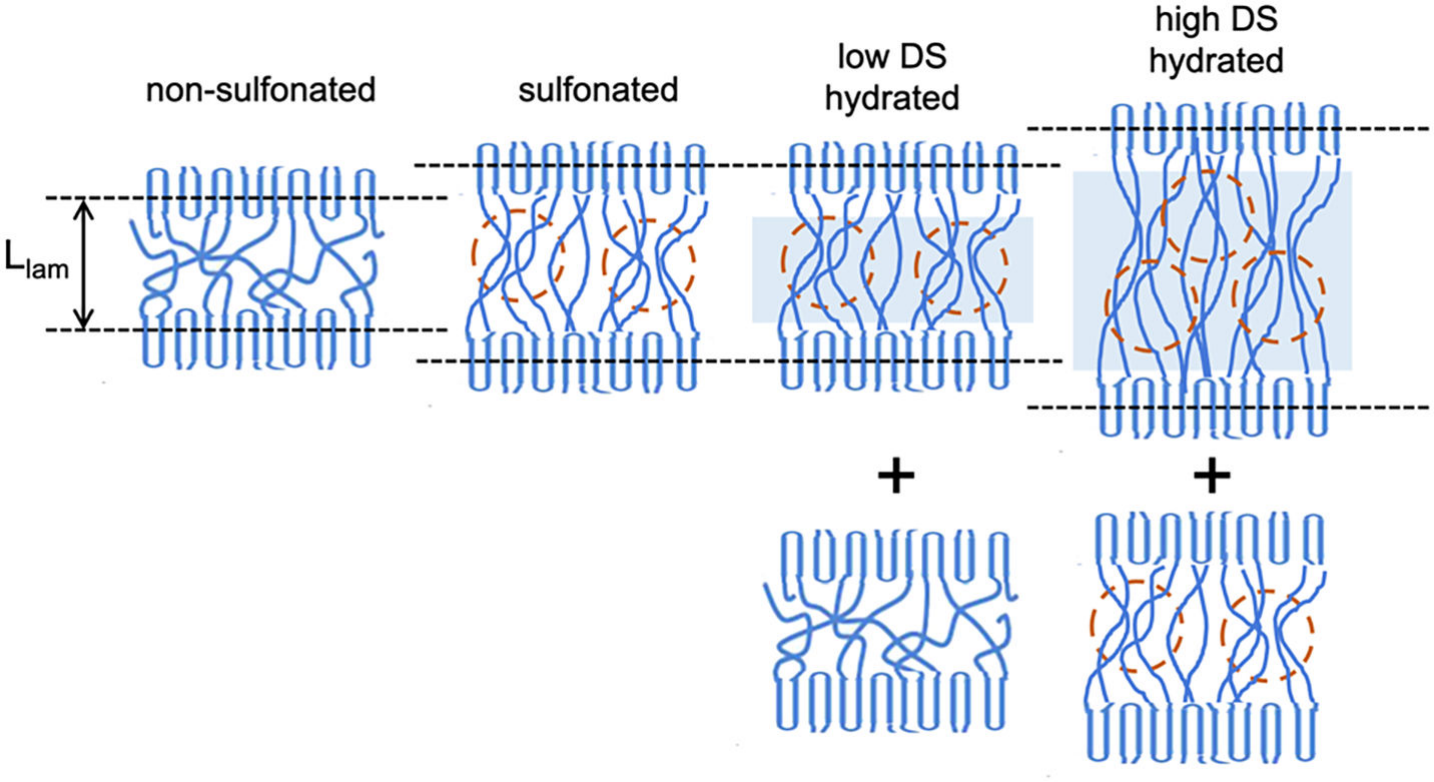
Schematic representation of the effects was induced by sulfonation and hydration of membranes with variable sulfonation degree DS. Sulfonation leads to the formation of ion clusters (areas marked in brown) and to an increased *L*_lam_ spacing, while membranes with low DS still contain nonsulfonated amorphous areas, as evidenced by the hydration of only the hydrophilic regions; membranes with high DS still contain dry ion clusters and highly hydrated areas at a lower degree of hydration than the equilibrated state in water, which are characterized by further swelling of *L*_lam_.

Structural analysis focused on characterizing the crystalline region of uniaxially deformed film samples, with emphasis on the scattering due to the interlamellar correlation effects occurring in oriented lamellar stacks. For this, only data collected over detector sectors parallel to the membrane deformation axis were considered. Scattered data were interpreted in real space using the one‐dimensional correlation function Γ_1_(*x*). The structural analysis revealed that sulfonation only affects the amorphous phase of the membranes and induces swelling of the long spacing. Hydration therefore also affects the functionalized amorphous regions. When working with deuterated sPS films, hydration with H_2_O enhances the contrast between the hydrated domains and the crystalline lamellae. On the other hand, the use of D_2_O in membranes with a low degree of sulfonation allows the correlation effects between oriented lamellae with interlamellar hydrated layers to be matched out. Only the correlation effects occurring in the nonsulfonated domains were visible in this case. In highly sulfonated membranes, the H_2_O contrast condition reveals a significant swelling of the hydrated interlamellar layers. On the other hand, for this kind of membranes only the correlation effects in the lamellar stacks containing still nonhydrated interlamellar layers are observed when D_2_O is used. The combined SANS experiment in different contrast conditions and the variable degree of sulfation of the membranes led to the conclusion that the sulfonation reaction takes place homogeneously across the entire amorphous phase of the films. Figure 10 summarizes the most important results of the current analysis.

## Conflicts of interest

The authors declare no conflicts of interest.
